# Where Some Nuts Come from

**Published:** 1890-03

**Authors:** 


### WHERE SOME NUTS COME FROM.

The most important item in the nut business is walnuts. Formerly these came almost entirely from Bordeaux, because at that time the affiliations of New York trade in this branch were with France. But the French walnut is comparatively a poor article ; the kernel is apt to be shrunken in the shell; the latter is often fiendishly hard, as well the youthful reveller knows, and the flavor of the meat is inferior. The Chicago houses buy perhaps one-tenth of the yearly stock from Bordeaux, a trifling matter of 10,000 or 12,000 bags. The very best come from Naples, for the nuts are the cleanest, and the kernels are full and of fine flavor. Several houses in the trade buy 2,000 or 3,000 cases each of walnuts from beautiful Naples yearly, and the traffic is constantly increasing. Next in rank come the California walnuts particularly those of the Las Nietas district, and so much are these liked that a single firm received twenty carloads at a consignment. The Brazil nut, which resembles the meat of the cocoanut, but is very much finer, is popular in the United States. All the Brazil nuts come from Rio Janeiro.

There was a time when a Christmas dessert was composed of almonds, raisins, figs and filberts, which the French called the four beggars, because no one would touch them—that is, among the adults. To the young Americans almonds and raisins are as dear as ever, and probably to the young French people also. The great rival of California is Spain, and the almonds of Tarragona are exported in considerable amounts. Both of these go to the husking-house, for the bakers and pastry-cooks who use almonds in large quantities prefer to buy them ready for use. Those who have eaten German almond cakes must confess that they are almost as delicious as macaroons, which are nearly all almond. The shelled almonds come to us direct from Italy and through London from the Valley of the Jordan. We get very little of anything from Palestine, but almonds we do get, and they are as good as any that come to the trade. But they have not acquired the art which the Californias have picked up from the Spaniards of making their boxes beautiful with showy.colored lithographs, and their boxes are plain, substantial receptacles, nothing more.

